# Supplementary material for: How does inquisitiveness matter for generativity and happiness?
Source: PLoS One. 2022 Feb 25;17(2):e0264222. doi: 10.1371/journal.pone.0264222 (PMC8880940; doi:10.1371/journal.pone.0264222)
Supplement: Attachment — Submitted filename: Ref1_PO_Inquisitiveness_December_25_2021.pdf [file pone.0264222.s002.pdf]

Our responses to reviewer 1 regarding the manuscript "How does inquisitiveness matter for generativity and happiness?" submitted to *PLOS ONE*

First, thank you for spending your time on our manuscript and providing us with your comments. Your comments are very constructive and useful for the improvement of our paper. We have tried to clarify all of your quarries and modified our manuscript, accordingly. Our responses to your comments are given below.

Reviewer 1's general comments and our responses

1. **Reviewer comment**: In this manuscript, the Authors aimed to How does inquisitiveness matter for generativity and happiness? The manuscript is well designed and written.

   Our response: We really appreciate your nice comments.

2. **Reviewer comment**: I would add a short comment in the Discussion section to analyze if the data collected are in line with similar investigations from western and other Asian countries. This would provide an idea about cultural differences, psychological issues, and lifestyle differences on inquisitiveness.

   Our response: Thank you for your comment. We conjecture that the results associated with inquisitiveness would hold when a similar study is conducted in other places. In fact, we have already started such a project in other countries. In the revised manuscript, we add such a short comment in the Discussion section. The modifications are made as follows:

   [***Page 14, lines 411-425***: *We note some limitations of our research and directions for future research. It should be noted that Japanese cross-sectional data are collected, utilized and analyzed in this study, excluding very young people. Further research shall be conducted to confirm the robustness of our results by spanning such young people or by considering different societies, such as western or other Asian countries, providing some insight about age and cultural differences. At this point in time, we conjecture that inquisitiveness remains consistent and important even in different ages and countries, being in line with our study. Moreover, as some studies have argued, it shall be desirable to collect and examine panel data to confirm and generalize our findings [13, 51, 52]. To this end, experimental methods can be employed to collect panel data and examine the possible causality among inquisitiveness, generativity and happiness. These caveats notwithstanding, it is our belief that this research is an essential first step toward understanding the importance of inquisitiveness along with generativity and happiness, hoping that further studies will ensure to identify how to enhance people's happiness and sustainability of societies.*] ■

3. **Reviewer comment**: I suggest shortening the introduction.

   Our response: Thank you for your comment. In accordance with your suggestion, we shorten the main texts of 113 words or so in the introduction section, shrinking to a total of 895 words. This has been achieved by changing the style of expressions, deleting unnecessary texts and moving some information to the footnote. However, considering the other reviewer's comments, we would like to retain some statements and structure of the introduction section in our manuscript, hoping your understanding. In the end, the introduction section is modified as follows:

[***Pages 1-3, lines 2-84***: *Curiosity and acceptance are important elements for one person to gain creativity, fulfillment and views [39, 71, 38, 28]. A child's tendency to ask a question shall be an initial step of building human relations and learning various things. In the literature, such a tendency is conceptualized as "inquisitiveness" representing curiosity & acceptance to something and someone different [41, 26]. Frazier et al. [25] examine adult-child conversational exchanges by focusing on young children's questions and adult's answers, claiming that such communications provide important bases for children's future life, especially regarding how they are able to grow through human interactions. Moreover, it is established that having and keeping nice relationships with family, friends and general others contribute to generativity and happiness [54, 33, 31, 68, 76]. Given this state of affairs, individual tendencies to be curious about and/or accept something and someone different (or new) may be a main engine for one person to be not only interactive with people in different generations but also happy. Therefore, this research addresses the role of inquisitiveness for generativity and happiness.*

Erikson [23] introduces the concept of generativity and defines it as a concern regarding the establishing and guiding of future generations in the life-span theory of personality development. Generativity is expressed in bearing and raising children but is by no means limited to the domain of parenthood [54]. Various activities and behaviors concerning future generations for helping and teaching something useful and interesting to young generations, are also considered expressions of generativity [56, 73]. Some scales of generativity have been developed to quantify such people's activities, behaviors and concerns, e.g., the Loyola generativity scale (LGS) and the generative behavior checklist (GBC) [54, 55, 53, 31]. Utilizing these scales, previous studies have characterized generativity concerning psychological and sociodemographic factors, such as aging, education, gender, health, income, marital status, political view, type of societies and value orientation [62, 64, 42, 65, 35, 68, 73]. Overall, it is established that age, marital status and type of societies are main determinants of generativity.

Happiness is taken to be a term to represent an outcome of a "good life," where people act and behave to seek happiness [58, 36, 47, 27].[1] *Maslow proposes a theory based on psychological needs and gratification processes, suggesting that people are happy as they become wealthy, i.e., Maslow's hypothesis. To examine this hypothesis [50], several researchers have developed and refined the measurements, such as the subjective happiness scale (SHS) and satisfaction with life scale (SWLS) [see, e.g., 16, 17, 48]. Veenhoven [75] and Diener and Diener [15] examine the hypothesis with cross-country level data utilizing happiness scales and conclude that wealth can account for variation in happiness across countries to a certain extent; however, there should be some other important predictors. Following these works, the literature has focused on how happiness is associated with cultural, sociodemographic and personal factors, other than wealth or income, including education, gender, marital status, self-esteem, human relations, optimism and extraversion [19, 20, 37, 44, 34, 61, 18, 11]. Overall, it is established that aging, income, human relationships, personality traits and value orientations matter for characterizing happiness [80, 82, 46, 7, 57, 49, 3].*

Previous studies have examined the relationship between generativity and happiness, often

[1] In this paper, we interchangeably use the term "wellbeing" to refer to "happiness."

along with social preferences [21, 31, 2, 43, 22, 66, 1, 59, 73, 70]. Aknin et al. [2] conduct survey experiments with 51 students of the University of British Columbia, claiming that social preferences are positively associated with happiness and there exists a positive feedback loop between the two. Timilsina et al. [73] compare prosociality and generativity between rural and urban people by conducting survey experiments in Nepal. They find that rural people are more prosocial and generative than urban ones, and claim that prosocial orientation shall contribute to generativity.[2] *They establish that generativity is a robust and consistent predictor of happiness, controlling for prosociality and some other key sociodemographic factors in the analyses. Overall, these studies suggest that generativity and prosociality can influence happiness [4, 33, 31, 14, 72].*

Inquisitiveness is a concept to represent curiosity & acceptance of something and someone different and/or new, and those with inquisitiveness tend to start communications with others by asking questions [30, 8, 6, 78, 79]. After development of the scales for inquisitiveness by Facione et al. [24], Hirayama and Kusumi [30] and Hogan and Hogan [32], some studies have been conducted to address how an inquisitive person behaves in terms of learning from and engaging with people regardless of their backgrounds, positions and roles as well as how such behaviors may lead to creative problem solving for nursing and schooling [81, 40, 32, 6, 69]. Hirayama and Kusumi [30] conduct questionnaire surveys with 426 Japanese university students and analyze the critical thinking attitudes on the process of drawing a conclusion. They find that inquisitiveness is an essential factor in reaching a conclusion not bounded by people's beliefs.[3] *Overall, inquisitiveness is a powerful source of engines that increases the motivation and behaviors in some situations, triggering people's communications with others and their interactions with unfamiliar environments [9, 5, 8, 12].*

No previous works have addressed how generativity and happiness are characterized by inquisitiveness, while both of these concepts are known to be highly concerned with how people build and keep relationships with family, friends and general others. Inquisitiveness is considered an important factor to trigger communications, being conjectured to contribute to maintaining nice human relations. Therefore, we hypothesize that inquisitiveness is an important determinant of happiness and generativity, empirically examining the relationships along with noncognitive, cognitive and sociodemographic factors in a single analytical framework. To this end, we conduct questionnaire surveys with 400 Japanese subjects to collect data, following previous studies that analyze the relationship between behaviors and happiness with cross-sectional data [74, 77, 45, 63, 67].[4] *With this data, our research addresses the following two open questions. (1) Does inquisitiveness play a role in generativity? (2) How does inquisitiveness, along with generativity, affect people's happiness?*]
■

[2]Building upon Timilsina et al. [73], Shahen et al. [70] conduct similar types of survey experiments in rural and urban areas of Bangladesh, collecting data on happiness and generativity along with prosociality and other variables.

[3]Nakagawa [60] also demonstrates that inquisitiveness is positively correlated with how people are well prepared for possible future disasters by conducting questionnaire surveys in Japan. Another group of studies analyze the role of inquisitiveness in leadership studies at schools and workplaces, generally confirming its importance in experiments and the fields [29, 10, 6, 79].

[4]There are several studies that apply cross-sectional data analyses, such as mediation analysis and regressions, to examine the relationships among personality traits, behaviors and happiness [74, 77, 67].

Bibliography

1. Aknin, L., Broesch, T., Hamlin, J., and Vondervoort, J. (2015). Prosocial behavior leads to happiness in a small-scale rural society. *Journal of experimental psychology*, 144:788–795.

2. Aknin, L., Dunn, E., and Norton, M. (2012). Happiness runs in a circular motion: Evidence for a positive feedback loop between prosocial spending and happiness. *Journal of happiness studies*, 13:347–355.

3. Au, A., Lai, S., Wu, W., Hofer, J., Busch, H., Šolcová, I., Tavel, P., and Cheng, S.-T. (2020). Generativity and positive emotion in older adults: Mediation of achievement and altruism goal attainment across three cultures. *Journal of happiness studies*, 21:677–692.

4. Aubin, E. and McAdams, D. (1995). The relations of generative concern and generative action to personality traits, satisfaction/happiness with lifea nd ego development. *Journal of adult development*, 2:99–112.

5. Baldwin, D. and Moses, L. (1996). The ontogeny of social information gathering. *Child development*, 67:1915–1939.

6. Bardone, E. and Secchi, D. (2017). Inquisitiveness: Distributing rational thinking. *Team performance management*, 23:66–81.

7. Bibi, F., Chaudhry, A., and Awan, E. (2015). Impact of gender, age and culture on life satisfaction. *Science international*, 27:1649–1652.

8. Black, S. (2005). The mindset of global leaders: Inquisitiveness and duality. In *Advances in global leadership*, pages 181–200. Emerald group publishing.

9. Blank, S. and Covington, M. (1965). Inducing children to ask questions in solving problems. *Journal of educational research*, 59:21–27.

10. Blickle, G., Meurs, J., Wihler, A., Ewen, C., and Peiseler, K. (2014). Leader inquisitiveness, political skill and follower attributions of leader charisma and effectiveness: Test of a moderated mediation model. *International journal of selection and assessment*, 22:272–285.

11. Chitchai, N., Senasu, K., and Sakworawich, A. (2018). The moderating effect of love of money on relationship between socioeconomic status and happiness. *Kasetsart journal of social sciences*, 2:1–9.

12. Cluver, A., Heyman, G., and Carver, L. (2013). Young children selectively seek help when solving problems. *Journal of experimental child psychology*, 115:570–578.

13. Cole, D. and Maxwell, S. (2003). Testing mediational models with longitudinal data: Questions and tips in the use of structural equation modeling. *Journal of abnormal psychology*, 112:558–577.

14. Cox, K., Wilt, J., Olson, B., and McAdams, D. (2010). Generativity, the big five and psychosocial adaptation in midlife adults. *Journal of personality*, 78:1185–1208.

15. Diener, E. and Diener, M. (1995). Cross-cultural correlates of life satisfaction and self-esteem. *Journal of personality and social psychology*, 68:653–663.

16. Diener, E., Emmons, R., Larsen, R., and Griffin, S. (1985). The satisfaction with life scale. *Journal of personality assessment*, 49:71–75.

17. Diener, E., Oishi, S., and Lucas, R. (2003). Personality, culture, and subjective well-being: Emotional and cognitive evaluations of life. *Annual review of psychology*, 54:403–425.

18. Diener, E. and Ryan, K. (2009). Subjective well-being: A general overview. *South African journal of psychology*, 39:391–406.

19. Diener, E., Sapyta, J., and Suh, E. (1998). Subjective well-being is essential to well-being. *Psychological inquiry*, 9:33–37.

20. Diener, E., Suh, E., Lucas, R., and Smith, H. (1999). Subjective well-being: Three decades of progress. *Psychological bulletin*, 125:276–302.

21. Dunn, E., Aknin, L., and Norton, M. (2008). Spending money on others promotes happiness. *Science*, 319:1687–1688.

22. Dunn, E., Aknin, L., and Norton, M. (2014). Prosocial spending and happiness: Using money to benefit others pays off. *Current directions in psychological science*, 23:41–47.

23. Erikson, E. (1963). *Childhood and society*. Norton, second edition.

24. Facione, P., Facione, N., and Giancarlo, A. (1992). *Test manual: The California critical thinking dispositions inventory*. Millbrae, CA.

25. Frazier, B., Gelman, S., and Wellman, H. (2009). Preschoolers' search for explanatory information within adult-child conversation. *Child development*, 80:1592–1611.

26. Fusaro, M. and Smith, M. (2018). Preschoolers' inquisitiveness and science-relevant problem solving. *Early childhood research quarterly*, 42:119–127.

27. Gilbert, A., Colley, K., and Roberts, D. (2016). Are rural residents happier? A quantitative analysis of subjective wellbeing in Scotland. *Journal of rural studies*, 44:37–45.

28. Hagtvedt, L., Dossinger, K., Harrison, S., and Huang, L. (2019). Curiosity made the cat more creative: Specific curiosity as a driver of creativity. *Organizational behavior and humen decision processes*, 150:1–13.

29. Harris, H. (2011). Inquisitiveness and abduction, Charles Peirce and moral imagination. *Business and professional ethics journal*, 30:293–305.

30. Hirayama, R. and Kusumi, T. (2004). Effect of critical thinking disposition on interpretation of controversial issues: Evaluating evidences and drawing conclusions. *Japanese journal of educational psychology*, 52:186–198.

31. Hofer, J., Busch, H., Chasiotis, A., Kärtner, J., and Campos, D. (2008). Concern for generativity and its relation to implicit pro-social power motivation, generative goals and satisfaction with life: A cross-cultural investigation. *Journal of personality*, 76:1–30.

32. Hogan, R. and Hogan, J. (2007). *The Hogan personality inventory*. Hogan Assessment Systems, third edition.

33. Huta, V. and Zuroff, D. (2007). Examining mediators of the link between generativity and well-being. *Journal of adult development*, 14:47–52.

34. Jan, M. and Masood, T. (2008). An assessment of life satisfaction among women. *Studies on home and community science*, 2:33–42.

35. Jones, B. and McAdams, D. (2013). Becoming generative: Socializing influences recalled in life stories in late midlife. *Journal of adult development*, 20:158–172.

36. Jorgensen, B., Jamieson, R., and Martin, J. (2010). Income, sense of community and subjective well-being: Combining economic and psychological variables. *Journal of economic psychology*, 31:612–623.

37. Kahneman, D., Diener, E., and Schwarz, N. (1999). *Well-being: Foundations of hedonic psychology*. Russell Sage foundation.

38. Kashdan, T., Afram, A., Brown, K., Birnbeck, M., and Drvoshanov, M. (2011). Curiosity enhances the role of mindfulness in reducing defensive responses to existential threat. *Personality and individual differences*, 50:1227–1232.

39. Kashdan, T., Gallagher, M., Silvia, P., Winterstein, B., Breen, W., Terhar, D., and Steger, M. (2009). The curiosity and exploration inventory-II: Development, factor structure, and psychometrics. *Journal of research in personality*, 43:987–998.

40. Kawashima, A. and Petrini, M. (2004). Study of critical thinking skills in nursing students and nurses in Japan. *Nurse education today*, 24:286–292.

41. Lagattuta, K. and Wellman, H. (2002). Differences in early parent-child conversations about negative versus positive emotions: Implications for the development of psychological understanding. *Development psychology*, 38:564–580.

42. Lawford, H., Pratt, M., Hunsberger, B., and Mark, P. (2005). Adolescent generativity: A longitudinal study of two possible contexts for learning concern for future generations. *Journal of research on adolescence*, 15:261–273.

43. Layous, K., Nelson, K., Oberle, E., Schonert-Reichl, K., and Lyubomirsky, S. (2012). Kindness counts: Prompting prosocial behavior in preadolescents boosts peer acceptance and well-being. *PLoS ONE*, 7:e51380.

44. Lee, D., Park, S., Uhlemann, M., and Patsult, P. (2000). What makes you happy? : A comparison of self-reported criteria of happiness between two cultures. *Social indicators research*, 50:351–362.

45. Lee, M.-A. and Kawachi, I. (2019). The keys to happiness: Associations between personal values regarding core life domains and happiness in South Korea. *PLoS ONE*, 14:e0209821.

46. Leung, A., Kier, C., Fung, T., Fung, L., and Sproule, R. (2011). Searching for happiness: The importance of social capital. *Journal of happiness studies*, 12:443–462.

47. Luhmann, M., Schimmack, U., and Eid, M. (2011). Stability and variability in the relationship between subjective well-being and income. *Journal of rural studies*, 45:186–197.

48. Lyubomirsky, S. and Lepper, H. (1999). A measure of subjective happiness: Preminary reliability and construct validation. *Social indicators research*, 46:137–155.

49. Magnani, E. and Zhu, R. (2018). Does kindness lead to happiness? Voluntary activities and subjective well-being. *Journal of behavioral and experimental economics*, 77:20–28.

50. Maslow, A. (1954). *Motivation and personality*. United States of America: Harper & Row Publisher.

51. Maxwell, S. and Cole, D. (2007). Bias in cross-sectional analyses of longitudinal mediation. *Psychological methods*, 12:23–44.

52. Maxwell, S., Cole, D., and Mitchell, M. (2011). Bias in cross-sectional analyses of longitudinal mediation: Partial and complete mediation under an autoregressive model. *Multivariate behavioral research*, 46:816–841.

53. McAdams, D. (2001). Generativity in midlife. In *Handbook of midlife development*, pages 395–443. John Wiley & Sons.

54. McAdams, D. and Aubin, E. (1992). A theory of generativity and its assessment through self-report, behavioral acts and narrative themes in autobiography. *Journal of personality and social psychology*, 62:1003–1015.

55. McAdams, D., Aubin, E., and Logan, R. (1993). Generativity among young, midlife and older adults. *Psychology and aging*, 8:221–230.

56. McAdams, D. and Logan, R. (2004). What is generativity? In *The generative society: Caring for future generations.*, pages 15–31. American psychological association.

57. Meisenberg, G. and Woodley, M. (2015). Gender differences in subjective well-being and their relationships with gender equality. *Journal of happiness studies*, 16:1539–1555.

58. Mentzakis, E. and Moro, M. (2009). The poor, the rich and the happy: Exploring the link between income and subjective well-being. *Journal of socio-economics*, 38:147–158.

59. Morselli, D. and Passini, S. (2015). Measuring prosocial attitudes for future generations: The social generativity scale. *Journal of adult development*, 22:173–182.

60. Nakagawa, Y. (2016). Effect of critical thinking disposition on household earthquake preparedness. *Natural hazards*, 81:807–828.

61. Oishi, S. and Diener, E. (2009). Goals, culture and subjective well-being. In *Culture and well-being*, pages 93–108. Springer.

62. Peterson, B. and Duncan, L. (1999). Generative concern, political commitment and charitable actions. *Journal of adult development*, 6:105–118.

63. Pluut, H. and Wonders, J. (2020). Not able to lead a healthy life when you need it the most: Dual role of lifestyle behaviors in the association of blurred work-life boundaries with well-being. *Frontiers in psychology*, 11:607294.

64. Pratt, M., Danso, H., Arnold, M., Norris, J., and Filyer, R. (2001). Adult generativity and the socialization of adolescents: Relations to mothers' and fathers' parenting beliefs, styles, and practices. *Journal of personality*, 69:89–120.

65. Rittenour, C. and Colaner, C. (2012). Finding female fulfillment: Intersecting role-based and morality-based identities of motherhood, feminism and generativity as predictors of women's self satisfaction and life satisfaction. *Sex roles*, 67:351–362.

66. Rudd, M., Aaker, J., and Norton, M. (2014). Getting the most out of giving: Concretely framing a prosocial goal maximizes happiness. *Journal of experimental social psychology*, 54:11–24.

67. Salavera, C., Usán, P., and Teruel, P. (2020). The mediating role of positive and negative affects in the relationship between self-esteem and happiness. *Psychology research and behavior management*, 13:355–361.

68. Schoklitsch, A. and Baumann, U. (2012). Generativity and aging: A promising future research topic? *Journal of aging studies*, 26:262–272.

69. Secchi, D. and Adamsen, B. (2017). Organisational cognition: A critical look at the theories in use. In *Cognition beyond the brain*, pages 305–331. Springer.

70. Shahen, M., Shahrier, S., and Kotani, K. (2019). Happiness, generativity and social preferences in a developing country: A possibility of future design. *Sustainability*, 11:5256.

71. Silvia, P. and Kashdan, T. (2009). Interesting things and curious people: Exploration and engagement as transient states and enduring strengths. *Social and personality psychology compass*, 3:785–797.

72. Tabuchi, M., Nakagawa, T., Miura, A., and Gondo, Y. (2015). Generativity and interaction between the old and young: The role of perceived respect and perceived rejection. *The Gerontologist*, 55:537–547.

73. Timilsina, R., Kotani, K., and Kamijo, Y. (2019). Generativity and social value orientation between rural and urban societies in a developing country. *Futures*, 105:124–132.

74. Tkach, C. and Lyubomirsky, S. (2006). How do people pursue happiness?: Relating personality, happiness-increasing strategies and well-being. *Journal of happiness studies*, 7:183–225.

75. Veenhoven, R. (1991). Is happiness relative? *Social indicators research*, 24:1–34.

76. Villar, F. (2012). Successful ageing and development: The contribution of generativity in older age. *Aging and society*, 32:1087–1105.

77. Warner, R. and Vroman, K. (2011). Happiness inducing behaviors in everyday life: An empirical assessment of "the how of happiness". *Journal of happiness studies*, 12:1063–1082.

78. Watson, L. (2018). Curiosity and inquisitivenes. In *The routledge handbook of virtue epistemology*, pages 155–166. Routledge.

79. Watson, L. (2019). Educating for inquisitiveness: A case against exemplarism for intellectual character education. *Journal of moral education*, 48:303–315.

80. Welsch, H. (2006). Environment and happiness: Valuation of air pollution using life satisfaction data. *Ecological economics*, 58:801–813.

81. Yeh, M.-L. (2002). Assessing the reliability and validity of the Chinese version of the California critical thinking disposition inventory. *International journal of nursing studies*, 39:123–132.

82. Zidansek, A. (2007). Sustainable development and happiness in nations. *Energy*, 32:891–897.
